# Mechanistic Insights into Protein Stability and Self-aggregation in GLUT1 Genetic Variants Causing GLUT1-Deficiency Syndrome

**DOI:** 10.1007/s00232-020-00108-3

**Published:** 2020-02-05

**Authors:** Mobeen Raja, Rolf K. H. Kinne

**Affiliations:** 1 Max Planck Institute of Molecular Physiology, Otto-Hahn-Strasse 11, 44227 Dortmund, Germany; 2 Algonquin College, 1385 Woodroffe Avenue, Ottawa, ON K2G 1V8 Canada

**Keywords:** Glucose transporter 1-deficiency syndrome, Sugar transport defect, Intermolecular interactions, Protein stability, Protein aggregation

## Abstract

Human sodium-independent glucose cotransporter 1 (hGLUT1) has been studied for its tetramerization and multimerization at the cell surface. Homozygous or compound heterozygous mutations in hGLUT1 elicit GLUT1-deficiency syndrome (GLUT1-DS), a metabolic disorder, which results in impaired glucose transport into the brain. The reduced cell surface expression or loss of function have been shown for some GLUT1 mutants. However, the mechanism by which deleterious mutations affect protein structure, conformational stability and GLUT1 oligomerization is not known and require investigation. In this review, we combined previous knowledge of GLUT1 mutations with hGLUT1 crystal structure to analyze native interactions and several natural single-point mutations. The modeling of native hGLUT1 structure confirmed the roles of native residues in forming a range of side-chain interactions. Interestingly, the modeled mutants pointed to the formation of a variety of non-native novel interactions, altering interaction networks and potentially eliciting protein misfolding. Self-aggregation of the last part of hGLUT1 was predicted using protein aggregation prediction tool. Furthermore, an increase in aggregation potential in the aggregation-prone regions was estimated for several mutants suggesting increased aggregation of misfolded protein. Protein stability change analysis predicted that GLUT1 mutant proteins are unstable. Combining GLUT1 oligomerization behavior with our modeling, aggregation prediction, and protein stability analyses, this work provides state-of-the-art view of GLUT1 genetic mutations that could destabilize native interactions, generate novel interactions, trigger protein misfolding, and enhance protein aggregation in a disease state.

**Electronic supplementary material:**

The online version of this article (10.1007/s00232-020-00108-3) contains supplementary material, which is available to authorized users.

## Introduction

Genetic mutations affect protein folding by triggering aggregation, shifting conformational equilibria, or disrupting phase transitions. They can decrease the thermodynamic stability or at least disrupt protein folding pathways. Disease-associated alleles are more likely to target amino acids that stabilize the active and functionally folded form of a protein and therefore induce perturbed phenotypes of the protein with altered stability (Sahni et al. 2015). It has been long known that many human diseases are caused by genetic mutations that trigger protein misfolding and aggregation. The very first reported mutation (a glutamate to valine switch) found in hemoglobin β-chain was linked to trigger protein aggregation in sickle-cell anemia (Ingram 1957). Most common neurodegenerative diseases such as Parkinson’s disease, Alzheimer’s disease, or amyotrophic lateral sclerosis are thought to be linked with protein aggregations (Valastyan and Lindquist 2014). However, our understanding of the precise cause of such diseases remains unclear. Yet, the physiological roles of many aggregating proteins, including prion protein, α-synuclein, or amyloid beta are still unknown (Bendor et al. 2013; Castle and Gill 2017).

Membrane proteins have a tendency of structural transition of their polypeptide chains into aggregation-prone conformation in a lipid bilayer (Gorbenko and Trusova 2011). In this regard, hydrogen-bonding, electrostatic, and hydrophobic interactions play major roles in initiating and modulating the protein misfolding and aggregation. However, mutations can disrupt important interactions or generate novel but redundant interactions by linking other parts of the interaction network together. Such changes can result in loss of all interactions upon protein misfolding, leading to protein degradation (Sahni et al. 2015; Zhong et al. 2009).

Inactivating or loss-of-function (LOF) mutations of major facilitated diffusion glucose transporter 1 (GLUT1) lead to GLUT1-deficiency syndrome (GLUT1-DS), an autosomal dominant haploinsufficiency disorder, which results in a low glucose concentration in the cerebrospinal fluid and therefore lack of energy supply to the brain (Rotstein et al. 2010; De Vivo et al. 1991). The symptoms of GLUT1-DS include early-onset drug-resistant seizures, mild-to-severe developmental delay, and an acquired microcephaly. A wide range of LOF mutations, including multiple deletions, missense, and frame shift mutations, lead to either loss of GLUT1 function or disappearance at the cell surface in the GLUT1 has been demonstrated in hundreds of patients causing an early-onset and more severe symptoms (De Vivo et al. 1991; Wang et al. 2005; Klepper and Leiendecker 2007). However, the mechanism by which GLUT1 mutations could lead to the failure of a protein to remain in its native functional conformational state, and whether LOF mutations trigger protein misfolding or even aggregation is not known.

Considering the pathophysiological relevance, GLUT1 has been a major focus for structural and functional studies over the last few decades (Hediger et al. 2013; Shi 2013). Elevated expression levels of GLUT1 have been reported in several cancer types, making GLUT1 as an important predictive marker for tumorigenesis (Ramani et al. 2013; Kaira et al. 2014). The major breakthrough by advances in crystallography happened when the crystal structure of hGLUT1 was reported at 3.2 Å resolution by introducing two mutations N45T and E329Q in the full-length GLUT1 (Deng et al. 2014). Later, the full-length hGLUT1 was cocrystallized with inhibitors and captured in an inward-open conformation at a resolution of 2.9–3.0 Å (Kapoor et al. 2016). These structures not only provided a clear model for substrate interaction with residues in the sugar binding pocket but also defined the inhibitor binding pocket in the central sugar binding site.

The crystal structure of hGLUT1 now allows more precise mapping and mechanistic elucidation of disease-associated mutations in GLUT1. In this study, we examined the hGLUT1 crystal structure to investigate multiple amino acids that constitute *hotspots* of genetic mutations causing GLUT1-DS. The native interactions were modeled to confirm their roles in stabilizing the transporter conformation and function. The modeling of mutant side chains was carried out to examine packing of introduced residues in the local environment and to predict novel or non-native redundant interactions in TM regions or cytosolic ICH domain. Protein aggregation prediction tools (PASTA and DeepDDG servers) were utilized to construct aggregation free energy profiles of wild-type hGLUT1 and its mutants. These analyses set future studies to examine at a molecular level how GLUT1 mutants causing GLUT1-DS could trigger unfavorable protein folding, increasing protein aggregation and ultimately causing sugar transport defects.

## Materials and Methods

### Analysis of Genetic Variations for Identification of Native and Redundant Interactions

**Fig. 1:**
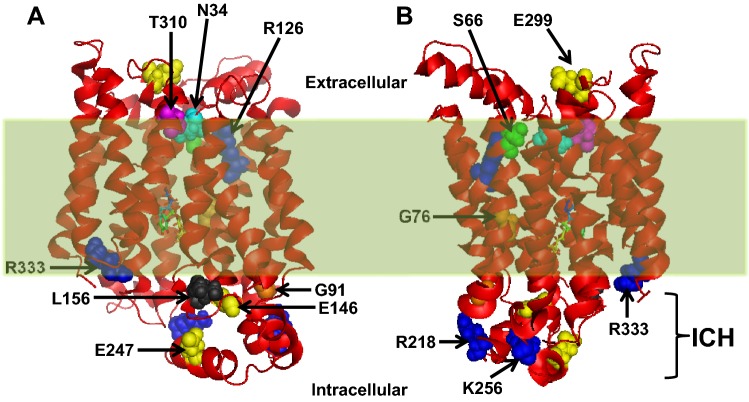
Three-dimensional structure of human GLUT1 (PDB ID: 5EQI) in the membrane plane depicting the positions of LOF pathogenic genetic mutations triggering GLUT-DS. The positions of intracellular helices (ICH) and several natural mutations (N34S, S66F, G76D, G91D, R126H/L, E146K, L156R/N, R218H, K256V, T310I, or R333W) in ball-and-stick model are highlighted. The structure was analyzed in a PyMol computer modeling program (http://www.pymol.org/). For clarity, two side views (**a** and **b**) are shown to depict the positions of genetic mutations with regard to the membrane plane (highlighted in light green)

For detection of native and non-native interactions, the crystal structure of hGLUT1 (PDB ID: 5EQI) was analyzed using PyMol modeling program (https://pymol.org/2/). Several natural missense mutations (N34S, S66F, G76D, G91D, R126H/L, E146K, L156R/N, R218H, K256V, T310I, or R333W), caused by single-nucleotide polymorphism (SNP), were specifically chosen for modeling of hGLUT1 (Fig. 1). Other mutations (i.e., addition or deletion mutations) were not feasible to predict confirmation/stability of the whole carrier using hGLUT1 PDB file. As shown in Fig. 1, the residues G91, E146, L156, R218, K256, and R333 are clustered on the intracellular side mainly within the large ICH domain of the transporter, whereas N34, S66, R126, E299, and T310 are positioned on the extracellular or TM regions. In PyMol, the distance (Å) between the donor–acceptor groups was measured using ‘measurement’ tool. For demonstration purposes, SI-Fig. 1 represents bond lengths (dotted lines) and distances (Å) for some residues, e.g., R126 (A), T310 (B), S66 (C), and G76D (D), to depict interactions among reactive groups in PyMol. The strengths of H-bonding (weak, moderate, or strong) were classified by the measured distances. The distance of 2.2–2.5 Å between the two side chains were considered strong, 2.5–3.2 Å as moderate, and 3.2–4.0 Å as weak interaction (Jeffrey 1997). For clarity and to avoid extensive labeling, all modeling figures were prepared without showing the measured bond distances that are compiled in Table 1. Mutagenesis tool was selected to introduce mutagenic residues and to depict the novel interactions among the donor–acceptor groups as described previously (Raja and Kinne 2012). The CaPTURE program was run (http://capture.caltech.edu/) using PDB ID: 5EQI to identify energetically significant cation–pi interactions within WT-hGLUT1 (Gallivan and Dougherty 1999).

**Table 1:** Summary of mutations and interaction partners exhibiting native or novel interactions

Mutation (SNPs)	Interacting amino acid	Measured distance (Å)	Interaction/H-bond	Type of interaction
N34S	N34‒S294 N34‒T295 N34‒T310	2.0 > 4.0 3.4	Strong H-bond Weak H-bond Weak H-bond	Native/non-covalent Native/non-covalent Native/non-covalent
S66F	S66‒Q422 S66F‒M420	2.6 –	Moderate H-bond Packing defect	Native/non-covalent –
G76D	G76D‒W412	1.7	Anion–quadrupole	Redundant/non-covalent
G91D	G91D‒F213	2.9	Anion–quadrupole	Redundant/non-covalent
R126H	R126‒N29 R126‒S68	2.0 2.2	Strong/covalent Strong/covalent	Native Native
R126L	R126L‒I33	3.8	Hydrophobic	Redundant/non-covalent
E146K	E146‒R92 E146K‒F213	2.1 3.3	Strong electrostatic Cation‒pi	Native/non-covalent Redundant/non-covalent
L156R	L156R	–	Packing defect	–
L156N	L156N‒S148	–	H-bond*	Redundant/non-covalent
R218H	R218‒E220 R218H‒F213	2.4 2.1	Strong electrostatic NH‒pi	Native/non-covalent Redundant/non-covalent
K256V	K256V-P401	3.6	Hydrophobic	Redundant/non-covalent
E299K	E299‒K38	1.9	Strong electrostatic	Native/non-covalent
T310I	T310‒N34 T310‒Q172	3.4 3.3	Weak H-bond Weak H-bond	Native/non-covalent Native/non-covalent
R333W	R333‒E329 R333W‒F389	> 4.0* 3.1	Flexible region* pi‒pi	Native/non-covalent Redundant/non-covalent

*Refers to the positioning of amino acids in dynamic loop which can change its position upon formation of interaction pairs

### Prediction of Protein Self-aggregation and Stability

The PASTA 2.0 server (http://protein.bio.unipd.it/pasta2/) predicts aggregation regions from protein sequences using a pairwise energy potential (Walsh et al. 2014). Amino acid sequences in FASTA format were used for prediction of self-aggregation in wild-type (WT)-GLUT1 and its genetic variants. The ‘mutate’ function was used to introduce single-point mutations and their effects on the aggregation ability of each protein sequence. As recommended by the server, energy threshold or cut-off was set to − 5 to produce 95% specificity of the server. The server predicted aggregation in energy units where 1 PASTA Energy Unit (PEU) was equivalent to 1.192 kcal/mol. The server computed the best aggregation pairing energy and the aggregating residues were assigned as parallel aggregating (P). The probability profiles and pairings (of aggregates) were calculated for positions of amino acids where *k* and *m* represent first and second amino acid in a pair, respectively. Linear probability profiles were also calculated by the server comparing aggregation and helix profile or aggregation and disorder profile as a function of the residue position or number (k).

More recently developed DeepDDG server (http://protein.org.cn/ddg.html), a neural network-based method (Cao et al. 2019), was used to predict the stability change of GLUT1 mutant proteins. This method predicts protein stability based on the solvent accessible surface area of the mutated residues as well as buried hydrophobic areas. As input file, PDB ID (5EQI) for hGLUT1 was provided and DeepDDG as network model was selected. Point mutations were entered to calculate the differences between Gibbs free energy (ΔΔG) of WT and mutant in order to predict the stability of GLUT1 mutants (kcal/mol), where predicted ΔΔG value > 0 represents stable mutant, and value < 0 an unstable mutant protein.

## Results

Human GLUT1 protein has been extensively studied biochemically and biophysically to understand its structure and function relationship (Hruz and Mueckler 2001; Mueckler et al. 1985). Similar to other major facilitator superfamily (MFS) transporters, GLUT1 was also considered to transport sugar as substrate by a mechanism similar to sodium-dependent glucose cotransporter (SGLT) family where substrate-binding site is alternately accessible from either side of the membrane through conformational changes of the transporters. Earlier investigations revealed that *SLC2A1* gene provides instruction for generating human GLUT1 protein consisting of 493 amino acids (Fukumoto et al. 1988). Sequence analysis combined with mutagenesis, topology studies, and the crystal structure illustrated that GLUT1 comprises 12 transmembrane (TM) alpha-helical domains (Fig. 1) that are linked by extra- and intracellular loops with both N- and C-termini facing the cytosol (Hruz and Mueckler 2001; Mueckler et al. 1985; Kapoor et al. 2016). The crystal structures of hGLUT1 as well as xylose transporter *XylE* from *Escherichia coli* authenticated several previously described biochemical findings that indicated the crucial roles of residues Q161, R126, Q279, Q282, N317, T321, W65, W388, W412, and V165 for glucose transport activity (Salas-Burgos et al. 2004; Olsowski et al. 2000; Seatter et al. 1998). In addition, hGLUT1 structure confirmed the previous finding that the central channel is composed of TM helices 2, 4, 5, 7, 8, and 10. The internal segment of the transport pathway is lined by both hydrophilic and hydrophobic amino acids that provides perfect interaction for the glucose molecule. The highly conserved intracellular helices IC1, 2, 3, and 4, collectively called intracellular helices (ICH), in GLUT family interact with the cytosolic parts of the transmembrane domains mediating the inter-domain interactions (Deng et al. 2014; Kapoor et al. 2016).

Most GLUT1-DS causing mutations target polar or charged amino acids, which seem to have both structural and functional roles in stabilizing the transporter (Deng et al. 2014). Mutations close to or within the substrate-binding domain have been proposed to trigger reduced recognition of D-glucose and strikingly compromised transport activity (Sun et al. 2012; Madej et al. 2014). However, how genetic mutations, especially the ones that are located away from the transport pathway or localized in the cytosolic domains, disrupt the structural integrity of GLUT1 causing total loss of function is not well known.

### N34S, T310I, and S66F Mutations in Destabilization of Intermolecular Interactions

**Fig. 2:**
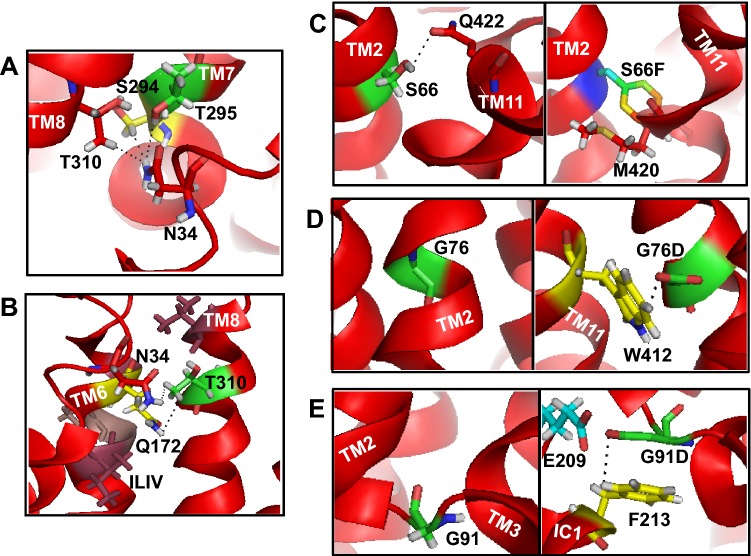
Modeling of N34 (**a**), T310 (**b**), S66 (**c**), G76 (**d**), and G91 (**e**) depicting their positioning, native, and novel interactions. The bond length distance, denoted by dotted lines, between the two side chains are summarized in Table 1. All structures were analyzed in a PyMol computer modeling program, as described in “Materials and Methods”

The missense N34S mutation due to SNP (280A>G) in exon II was reported in 2005 for the first time in two patients with GLUT1 deficiency syndrome (Wang et al. 2005). In the crystal structure of GLUT1, N34 is located on membrane localized topological domain linking TM1 and TM1e (Kapoor et al. 2016), where it was shown to organize the outer face of GLUT1 via H-bonding to S294 and T295 on TM7 and T310 on TM8 (Fig. 2a). The N34S mutation replaces asparagine, which is amino double polar amino acid, and adds single hydroxyl group of serine (an uncharged amino acid), hence destabilizing the organization center and exhibiting ~ 55% of WT glucose uptake activity (Wang et al. 2005). Table 1 illustrates the measured distances between the two side chains. In addition, the strength of H-bonds and type of interaction are summarized based on the distance between the donor–acceptor groups.

Next, we modeled T310I missense mutation which is involved in H-bonding to N34 within the same organizing center as described above. T310I is caused by a single-nucleotide mutation at 929 nucleotide position in exon VII (Klepper et al. 1999a). This mutation switches threonine to a bulky isoleucine and triggers sugar transport defect exhibiting 53% of WT-GLUT1 glucose uptake activity, but not trafficking defect, as studied by quantification of immunoreactive GLUT1 protein in erythrocyte membranes (Klepper et al. 1999b; Iserovich et al. 2002).

In the crystal structure, T310 is located on the extracellular side of the putative transport path (Fig. 2b) where it forms H-bonds with N34 and Q172 located on TM6 (Kapoor et al. 2016), as described above. An extended motif of serine/threonine hydroxyl groups has been shown to be important for oligomerization or helix–helix interactions (Dawson et al. 2002). Furthermore, mutation of T310 to a non-polar isoleucine would result in an increase in bulk affecting the packing of residues around the TM6 and TM8 and causing sugar transport defect. These notions are well supported by previous mutagenesis and functional studies revealing that the size and hydrophilicity of the side chain at position 310 (T310) are crucial in GLUT1 structure and function (Wang et al. 2003).

S66F mutation, an autosomal dominant mutation in exon III, is caused by substitution of serine with phenylalanine (Klepper et al. 1999b). S66, located on TM2, is in proximity to Q422 in TM11 and can favor a moderate H-bond formation between serine’s hydroxyl and glutamine’s amino group (Fig. 2c, Table 1). The crystal structure and modeling indicate that S66F mutation may not only perturbs helix–helix interaction by destabilizing S66-Q422 H-bond but also could interfere with the packing of TM2 and TM11 due to bulky nature of phenylalanine ring. Such modification in protein structure does not interfere with protein expression and/or targeting to the cell surface, however; a significantly reduced membrane association and an impaired glucose transport (45% of wild-type glucose uptake activity) explains the effect of structural instability (Klepper et al. 1999b).

### G76D and G91D Mutations Involved in Redundant Interactions

The genetic mutation G76D, a de novo mutation in exon III, is caused by SNP at position 227 (Tzadok et al. 2014). G76 is embedded in the TM2 which is positioned along the transport pathway (Fig. 2d, left side). Substitution of glycine (a particularly small, hydrophobic and neutral amino acid) to aspartate (a long, hydrophilic and negatively charged amino acid) has not been investigated for its adverse effects on GLUT1 transport function. However, the patient with this mutation was diagnosed with GLUT1-DS after exhibiting reduction in myoclonic absence seizures during a short fasting period related to a febrile seizures (Tzadok et al. 2014). The modeling of GLUT1 structure suggests that G76 is not a part of other transmembrane helices as its side chain is not energetically close enough to H-bond to another residue in the neighboring helix (e.g., TM11). However, G76D mutation (Fig. 2d, right side) would result in increase in bulk and further formation of strong, however, redundant anion–quadrupole interaction between aspartate 76 and tryptophan 412 located on TM11 (Table 1). Such H-bonding interactions are known to exist between certain anions (aspartate, glutamate, and phosphate) and aromatic amino acids (tryptophan, tyrosine, and phenylalanine) rings in protein–protein interfaces and membrane proteins (Chakravarty et al. 2018) with tryptophan exhibiting the highest tendency for this type of interaction.

G91D mutation is caused by SNP at nucleotide position 272 (GGC → GAC) in exon IV (Klepper et al. 2001). It is located on the loop linking TM2 and TM3 on the intracellular side where it does not seem to be involved in interaction with other residues (Fig. 2e, left side). According to the modeling, the side chain of G91D is located quite close to another negatively charged E209 that could trigger electrostatic repulsion (Fig. 2e, right side). This repulsion would be expected to bring G91D in a proximity to phenylalanine 213, which is located on IC1, to form redundant anion–quadrupole interaction between G91D and F213 (Table 1), similar to the effect described above for G76D mutation.

### R126H/L and L156R/N Mutations in Destabilizing Structure and Establishing Redundant Interactions

**Fig. 3:**
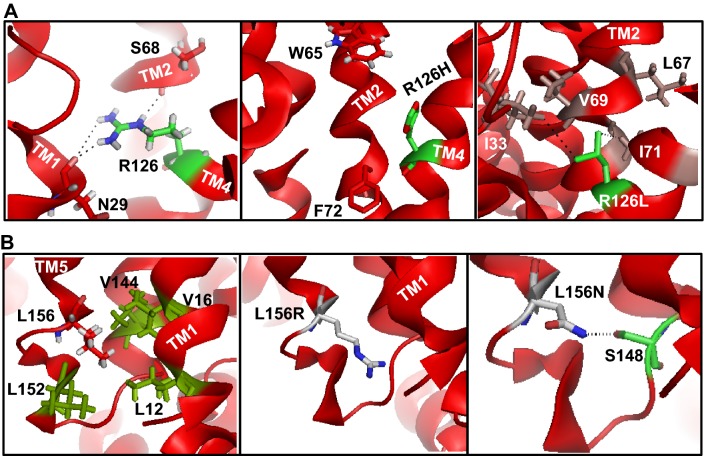
**a** Interaction of R126 (TM4) with N29 and S68 in TM1 and TM2, respectively. R126H may alter the conformational stability of TM4 and TM2. For R126L mutation, the introduced hydrophobic leucine may interact with another hydrophobic residues or may involve cluster formation via interaction between R126L, I71, and I33. **b** Modeling of L156, L156R, or L156N. In particular, L156N may cause conformational changes via L156N-S148 side-chain interaction. See Table 1

Both R126H and R126L mutations are caused by SNP in exon IV (Brockmann et al. 2001; Wang et al. 2000; Pascual et al. 2002) R126H, caused by SNP (556G>A), was reported in five patients and transport studies showed ~ 57% of WT-GLUT1 glucose uptake activity, further illustrating that the charge at this position is important in facilitation of glucose transport (Brockmann et al. 2001; Pascual et al. 2002). According to 3D-structure, R126 is located away from the transport path and buried in TM4 where it strongly interacts with the amino group of N26 and backbone of S68 in the adjacent TM1 and TM2, respectively (Fig. 3a, Table 1). Exchange of R126 by charged histidine would remove strong H-bonds and introduce aromatic imidazole causing an increase in bulk in the adjacent TM2 where aromatic W65 and F72 are localized.

**Fig. 4:**
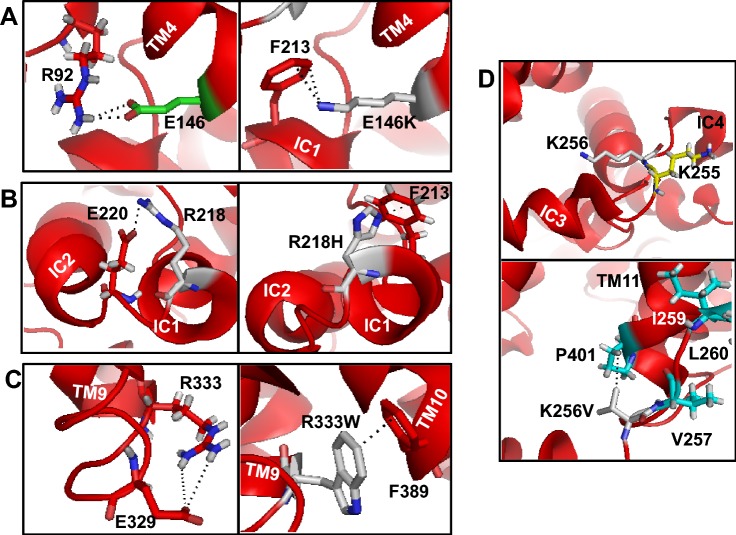
Illustration of electrostatic interactions between E146–R92 (**a**), R218–E220 (**b**), and R333–E329 (**c**), as summarized in Table 1. The right hand panels depict non-local interactions stabilizing distinct conformation. **d** Modeling of K256 in the central pore (upper panel). The K256V mutation is predicted to cause hydrophobic interaction (lower panel)

For R126L mutation (Wang et al. 2000), the substituted hydrophobic leucine seems to be positioned in the vicinity of another hydrophobic residue I33 in helix 1 (Fig. 4a). This could favor hydrophobic cluster formation among R126L, I33, V69, and I71. Such situation may alter helix–helix interaction and protein folding thereby impairing GLUT1 targeting (20% of native GLUT1) to the plasma membrane (Brockmann et al. 2001; Wang et al. 2000; Pascual et al. 2002). Furthermore, using 3-*O*-methyl-D-glucose as substrate of *Xenopus* oocytes, only 3.2% uptake was observed by the R126L mutant (Pascual et al. 2008).

L156R and L156N are novel mutations caused by SNP in exon IV (Tzadok et al. 2014). This mutant has not been studied biochemically and yet the transport function of L156R/N carrying GLUT1 protein is unknown. However, the patient with GLUT1-DS exhibited daily morning prolonged absences, other neurologic deficits, mild mental retardation, and microcephaly (Tzadok et al. 2014). In GLUT1 structure, L156 is located in TM5 where it is a part of a hydrophobic cluster comprising several hydrophobic residues L152, L12, V144, and V16 (Fig. 3b). L156R/N causes significant change as it switches hydrophobic leucine to positively charged non-hydrophobic arginine or uncharged polar asparagine. The modeling suggests that L156R side chain does not interact with any other amino acid(s) to form salt bridge; however, the removal of hydrophobic leucine and introduction of bulky side chain of arginine may alter the packing of neighboring IC1, 2, or 3 that are believed to maintain a proper conformation of the transporter (Kapoor et al. 2016). L156N mutation could probably pull S148 (located on the unordered or flexible loop) side-chain OH group to form favorable H-bonding. This redundant interaction could therefore cause a conformational change in this area, including IC1, 2, and 3, leading to protein misfolding and transport defect.

### Destabilizing Salt Bridges by E146K, R218H and R333W Mutations

Mutation E146K, caused by a SNP (GAA → AAA) in exon IV, is located on the intracellular side on TM4. Mutation of a negatively charged E146 to positively charged lysine leads to glucose transport defect (Wang et al. 2000). According to the crystal structure and our modeling, E146 is located quite close to R92 to establish strong electrostatic interaction between the oppositely charged side chains (Fig. 4a, Table 1), thereby stabilizing the conformation of GLUT1 in this region. However, E146K mutation removes this interaction and apparently establishes a redundant interaction between E146K and F213 (located on IC2) via cation–pi interaction. Both cation–pi (between cationic side chain of lysine/arginine and aromatic side chain of phenylalanine/tryptophan/tyrosine) and pi–pi interactions (between aromatic side chains) have been dictated by distance and angle between the reactive groups (Gallivan and Dougherty 1999). To validate whether cation–pi interactions can be detected in GLUT1, the CaPTURE program was run. Only 3 energetically significant native cation–pi interactions were predicted that involved pairs of Arg51/Phe119, Arg126/Phe72, and Arg333/Phe389 with E(electrostatic) between − 1.30 and − 1.10 kcal/mol and with E (van der Waals) between − 1.75 and − 1.30 kcal/mol. PyMol modeling was carried out for Arg126/Phe72 and Arg333/Phe389 to determine the location of interacting residues (SI-Fig. 2, A and B). Modeling depicts that the distances between the reactive groups of both Arg/Phe pairs are > 4.0 Å. However, close coupling of such pairs is quite possible to form cation–pi interactions when GLUT1 undergoes conformational change during transport cycle (Deng et al. 2014; Kapoor et al. 2016). Considering that the CaPTURE program is only useful for known protein structures, cation–pi interactions could not be predicted in E146K mutant, which seems to interact with F213.

Another missense mutation in GLUT1-DS is caused by SNP (653G>A) in exon V, causing arginine in position 218 to be replaced by histidine (R218H). It has been shown that R218H causing mild-to-severe symptomatic GLUT1-DS could be associated to the effect of either diverse epigenetic or modifier genes on GLUT1 protein function (Klepper et al. 2005). However, this mutation has not been characterized for protein mistrafficking or glucose transport defect.

R218 is located in cytoplasmic IC1 (between H6 and H7). The structure and modeling show that R218 is localized quite close to E220 (located on unstructured loop connecting IC1 and IC2) to form H-bond via strong ionic interaction (Table 1) stabilizing both ICs in their native states (Fig. 4b, left side). Replacing R218 by histidine introduces an aromatic imidazole ring removing strong electrostatic interaction. The introduced histidine is apparently localized in the vicinity to F213 within the same IC1 to form strong NH‒pi bond between imidazole and benzene ring (Fig. 4b, right side). These kinds of interactions are known as T-shaped histidine‒aromatic contacts that arise frequently among the amino acid side groups in proteins (Trachsel et al. 2015).

R333 is located on TM9 facing cytosol and forms ionic bond with E329 which is located on the loop preceding TM9 (Fig. 4c, right side). Missense mutation of R333 (R333W) to a non-polar tryptophan is due to a SNP (CGG → TGG) in exon VIII (Wang et al. 2000). This residue is highly conserved among members of the GLUT family and the MFS membrane transport proteins from *Archaebacteria* throughout humans (Wang et al. 2000). R333 is potentially involved in establishing the membrane topology of human GLUT1. However, R333W mutation causes severe defects in GLUT1 function thereby exhibiting only 43% of WT glucose uptake activity (Wang et al. 2005; Pascual et al. 2002). The importance of this position is conserved among other GLUT family members as a corresponding R380W mutation in GLUT9 leads to renal hypouricemia type-2 (Kawamura et al. 2011).

Removal of ionic interaction by R333W can destabilize the segment between TM8 and TM9 leading to improper conformation or misfolding. According to the modeling, R333W variant is positioned quite close to F389 which is located on TM10b (Fig. 4c, left side). This can favor redundant formation of tryptophan–phenylalanine pair via pi‒pi interaction (Table 1) with face-to-face geometry. Such aromatic–aromatic interactions are known to exist in proteins and can significantly contribute to stabilize the protein conformation (Ramnath et al. 2012; Cherumuttathu et al. 2009). The mutation R333W could have another possible effect due to the preferential positioning of the aromatic side chain of substituted tryptophan at the membrane–water interface (Özdirekcan et al. 2008), thereby altering the packing of TM9.

### K256V Mutation in Hydrophobic Interactions

Missense mutation K256 is caused by SNP (945A>G) in exon VI (Pascual et al. 2002). Site-directed mutagenesis studies in *xenopus* oocytes revealed mild decrease in GLUT1 protein expression (~ 85% of native GLUT1) with no clinical symptoms and 12.7% decrease in 3-*O*-methyl-D-glucose (3-OMG) uptake by the K256V mutant (Rotstein et al. 2010). However, major changes were observed in red blood cell uptake. The patient’s 3-OMG RBC uptake was ~ 35% as compared to control (100%). The genetic analysis revealed compound heterozygosity due to two heterozygous missense mutations, K256V and R126L in exon VI and IV, respectively (Rotstein et al. 2010).

In the crystal structure, K256 is located in the loop preceding IC4, a part of long ICH. The side chain of K256 does not seem to H-bond to any amino acid as it points toward the central cavity of GLUT1 (Fig. 4d, top panel). Interestingly, the loop preceding IC4 carries K255 and K256 that could interact with the negatively charged lipids on the cytoplasmic side and help stabilize IC4 at the membrane surface, similar to what has been depicted in other membrane proteins and their interaction with the negatively charged lipids (Raja et al. 2007; Raja 2010). Replacing K256 by a hydrophobic valine could weaken IC4-lipid interaction causing a shift in the short IC4 positioning with respect to the membrane surface and altering protein conformation. Furthermore, introduction of valine at 256 position would increase the hydrophobicity of IC4 also via possible hydrophobic interaction with P401 (located on TM11) which is a part of hydrophobic cluster (V257, I259, L260, and L262) in this region (Fig. 4d, bottom panel).

### Effect of Genetic Mutation on Free Energy and Protein Aggregation Potential

**Fig. 5:**
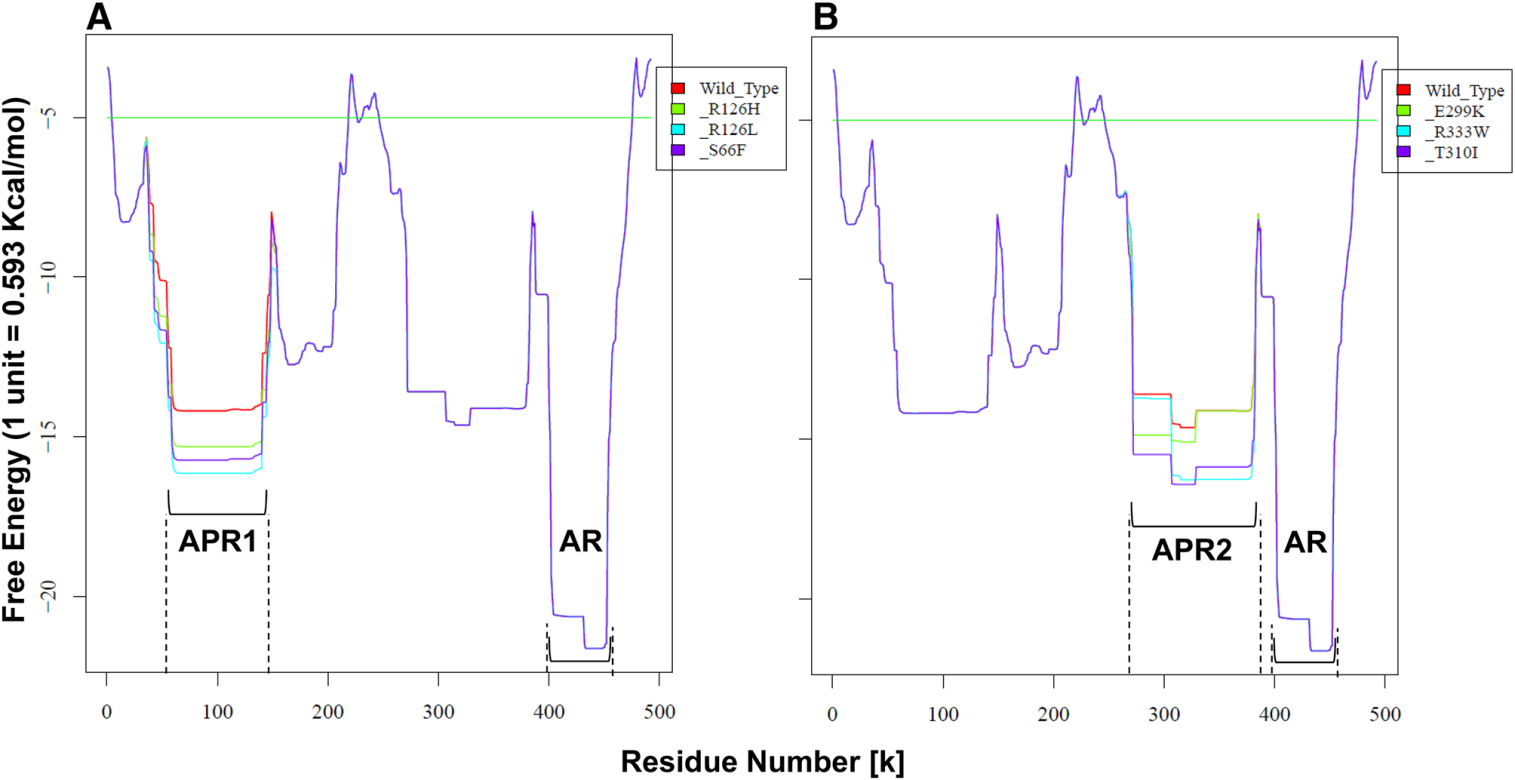
Aggregation free energy profiles of WT vs. S66F, R126H, R126L (**a**), and WT vs. E299K, T310I, and R333W (**b**). The APR1 and 2 represent aggregation-prone regions 1 and 2 that comprise of ~ 50–150 and ~ 270–390 residues, respectively. AR represents aggregating region, which corresponds to 402–452 residues

To support our modeling in prediction of novel interactions and protein misfolding, we used PASTA 2.0 server to detect protein aggregation in WT-GLUT1 and its genetic variants. The comparison of free energy profiles of full-length WT-GLUT1 and single mutants S66F, R126H, and R126L are shown in Fig. 5a. Compared to WT-GLUT1, lower energy (an increase in aggregation potential) of mutants was detected in the so-called aggregation-prone region 1 (APR1), as highlighted by the dotted lines. This region corresponds to residues ~ 50–150 that constitute TM2, 3, and 4. WT-GLUT1 exhibited lowest energy of − 14.2 kcal/mol of APR1. For mutants R126H, S66F, and R126L, aggregation potential of APR1 was increased due to a slight decrease in free energies by − 15.3, − 15.8, and − 16.3 kcal/mol, respectively. For mutants E299K, T310I, and R333W (Fig. 5b), an increase in aggregation potential was predicted in the other region, called aggregation-prone region (APR2), which corresponds to residues ~ 270–390 (or constitute TM7, 8, 9 and 10a). For WT-GLUT1, the best free energy of − 14.5 kcal/mol was estimated for APR2. The mutants E299K, R333W, and T310I exhibited best aggregation energy of − 15.1, − 16.4, and − 16.5, respectively. All other mutants did not exhibit any difference in free energy profile as compared to WT-GLUT1 (SI-Fig. 3).

**Fig. 6:**
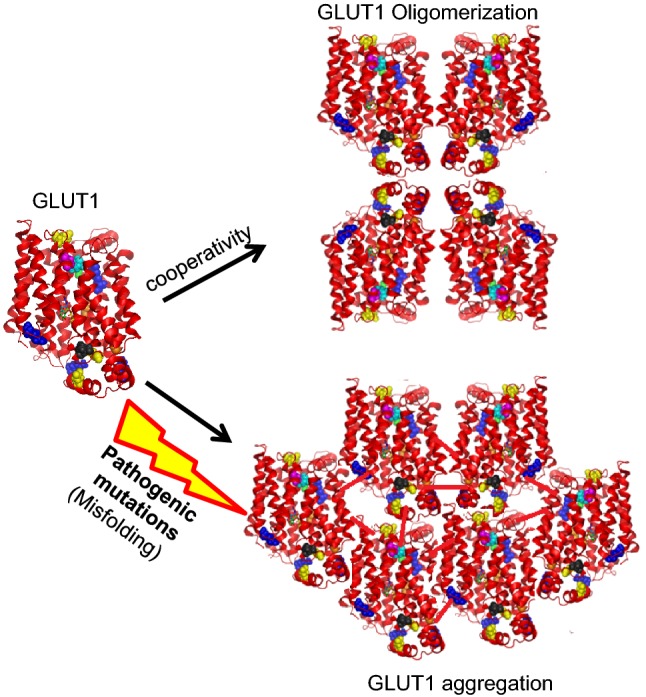
A hypothetical model of oligomerization of WT-GLUT1 and aggregation of misfolded mutant GLUT1. The cooperativity among GLUT1 subunits leads to tetramerization or oligomerization. However, destabilizing pathogenic mutations may elicit unfavorable conformation, misfolding, and aggregation in GLUT1-DS

Furthermore, the lowest energy was calculated for so-called aggregating region (AR), which was found to be similar among WT and all other mutants (Fig. 5a, b). This AR corresponds to residues 402–452 (highlighted in blue) as shown in the amino acid sequence of WT-GLUT1 (SI-Fig. 4). The prediction of parallel aggregates (P) and the probability of aggregation among pairs (denoted by *k* and *m*) are also represented. The helical prediction by PASTA 2.0 (TM1–TM12, highlighted in green) agrees well with GLUT1 structure and sequence. The pairing description of residues 402–452 for WT-GLUT1 is shown (SI-Fig. 5), illustrating the formation of 20 aggregates or pairing segments of varying sizes that occurred in the region between residues 402 and 452. The aggregation energy of AR (ranged between − 18.6 to − 21.2 kcal/mol) was found to be similar for WT and all mutants. The linear probabilities of aggregation vs. helix profile and aggregation vs. disorder profile were calculated, agreeing with the amino acid sequence-based aggregation prediction (SI-Fig. 6). In addition to identification of AR in all proteins, the aggregation prediction server also recognized aggregation-prone regions, such as APR1 and APR2. As predicted above, several genetic mutants exhibited minor differences in energy profiles as compared to WT that need to be taken into account when protein folding is significantly affected by small changes in the free energies (see “Discussion” section).

Using DeepDDG server, the ΔΔG values (kcal/mol) were found to be < 0 for the following mutants: N34S (− 0.586), S66F (− 0.703), G76D (− 2.625), G91D (− 2.716), R126H (− 1.861), E146K (− 1.183), L156R (− 1.874), L156N (− 1.280), R218H (− 0.467), K256V (− 0.632), T310I (− 1.047), and R333W (− 0.323), indicating unstable proteins, except for R126L (0.477) which probably behaved similar to WT. The predictions from both PASTA and DeepDDG programs indicate that genetic mutations destabilize the GLUT1 protein structure leading to unstable proteins or protein aggregation.

## Discussion

Three general effects were considered in the present study: (1) side-chain interactions which could influence ligand binding, rigid carrier movements and small gate motions, (2) folding changes that can result in misfolding, and (3) aggregation potential which informs our understanding of intra and intermolecular domain interactions. Our structural analyses of GLUT1 mutations not only predicted native stabilizing forces but also identified unique characteristics of mutated residues in establishing novel non-covalent interactions (especially hydrogen bonds, anion-quadrupole, cation‒pi, NH‒pi, pi‒pi, and hydrophobic interactions), thereby altering native GLUT1 structure and conformation. GLUT1 has been shown to undergo several functional conformations during transport cycle (Deng et al. 2014; Kapoor et al. 2016). Such transitioning can be energetically quite close to the native state of the transporter. However, LOF mutations may very well interfere with this equilibrium, leading to protein misfolding or significant loss of the active conformation (Hediger et al. 2013; Shi 2013; Deng et al. 2014).

Using theoretical prediction and simulation (Clementi and Plotkin 2004), it has been shown that in proteins non-native interactions exist, but they are too small to perturb the folding as compared to the large native interaction energies that drive native folding. If non-native interactions are increased to larger values, the folding rates are significantly decreased. Hence, the thermodynamic free energy barrier is lowered by the presence of non-native interactions triggering stable misfolded structures (Clementi and Plotkin 2004). There exists an energy gap between the native and any other potential conformations of a protein (Bryngelson et al. 1995; Karplus and Sali 1995), as observed among WT and some GLUT1 mutants in the APR1 or 2. Such gaps can be filled by native non-covalent interactions that could help stabilize the native conformation (Dill 1990). This is not possible when irreversible mutations destabilize crucial native interactions. In fact, we predicted that mutating residues in a protein not only destabilize the native conformation but also make the introduced incompatible residues interact with the neighboring residue and cause unfavorable non-local interactions in the tertiary structure, as summarized in Table 1. This step may be required when non-native conformation is urged to overcome an energy gap. However, such native-like redundant conformation may lead to a distinct or non-functional conformation.

A single mutation can significantly destabilize a protein by several kcal/mol, further lowering energy and leading to an unfolding or aggregation state (Tokuriki et al. 2008). Computational predictions have suggested that 20–50% of disease causing mutations decrease protein stability (Berliner et al. 2014; Yue et al. 2005). In particular, it was estimated that ~ 20% of genetic variants destabilize proteins by over 1.5 kcal/mol. Such energy difference corresponds to almost tenfold increase in the ratio of unfolded to folded protein (Berliner et al. 2014). In line with this information, the free energy lowered by 1.5–2.5 kcal/mol (Fig. 5) points to an unfolding or rather misfolding tendency of the APR1 or 2 in GLUT1 mutants. Such aggregation-prone regions represent dynamic structures and may undergo significant conformational changes during transport cycle (Deng et al. 2014; Kapoor et al. 2016). Perhaps misfolding of such regions could also interfere with GLUT1 structure determination, where binding of inhibitors within glucose binding region (Kapoor et al. 2016) might stabilize the protein structure by eliciting stabilizing interactions, improving protein solubility and stability for crystallization studies.

According to the modeling, the mutation S66F located on TM2 could destabilize the interaction with Q422 by introducing bulky phenylalanine (Fig. 2c), leading to misfolding or packing defect of TM2 and potential aggregation of APR1 (representing TM2, 3, and 4). Similarly, R126L/H located on TM4 would destabilize the network of interactions, involving TM1 and 2, by introducing bulky histidine or hydrophobic valine, thereby increasing aggregation potential of APR1. The mutation T310I (located on TM8) not only destabilizes the organizing center of the transporter (Fig. 2b) but could also lead to misfolding or potential aggregation of APR2 (TM7, 8, 9, and 10a). Furthermore, R333W (located on TM9) would be expected to elicit novel interaction with F389 on TM10 (Fig. 4c) triggering unfavorable conformational change and further increasing the aggregation potential of APR2.

However, a word of caution is also necessary. No changes in aggregation free energy profiles were observed for mutants N34S, G76D, G91D, E146K, L156R/N, R218H, and K256V using PASTA. Perhaps such predictions are limited due to the approximations used in the underlying algorithm that were too large to assess the relevance of these single mutations. Another explanation is that not all mutations may necessarily elicit protein aggregation. The prediction of structural changes followed by novel redundant interactions could also stabilize the non-native conformation of the protein leading to either impaired protein assembly in the membrane, sugar uptake or both. Nevertheless, the predictions from PyMol and PASTA need to be combined with experimental data using site-directed mutagenesis to generate single-point and double mutants carrying genetic mutation and mutation of predicted interacting partner, as calculated in our analyses. These mutants should be expressed in a suitable host (e.g., HEK cells or Xenopus oocytes) to determine their expression levels, transport activities, protein oligomerization, and aggregation detection based on size exclusion chromatography of detergent solubilized GLUT1 proteins (Mueckler and Makepeace 2005; De Zutter et al. 2013), and sensitivities to substrates or inhibitors, e.g., cytochalasin B or phenylalanine amides.

The calculated aggregation free energy profiles do not take into account distinct protein folding in the membrane environment. Yet, qualitatively similar shapes of aggregation free energy profiles suggest that WT and GLUT1 mutants exist in thermodynamically unfavorable conformation. This agrees well with the notion that proteins containing intrinsically disordered regions, including the WT proteins, are prone to misfolding (Qin et al. 2013). However, mutations further promote the aggregation of the protein. Hence, the phenotypic consequences of the mutations cannot be easily elucidated by the function of the WT protein, especially when the protein is known to exist in aggregates or oligomeric intermediates (De Zutter et al. 2013; Looyenga et al. 2016).

GLUT1 has been studied for its dimerization and tetramerization at the cell surface using GLUT1–GLUT3 chimeras. The tetramers were evaluated to have fourfold more catalytic activity than GLUT dimers suggesting that allosteric transport behavior is reflected by subunit interactions (De Zutter et al. 2013). This study also highlighted the roles of TM2, 5, 8, and 11 in GLUT1 dimerization, and TM9 in tetramerization. Interestingly, GLUT1 was later detected to behave as a multimeric complex in live cells where monomers were found to be initially associated via N-to-N-terminal dimers, which could then form tetramers stabilized by homomeric interactions between the adjacent C-termini of the four monomers (Looyenga et al. 2016). These studies suggest that tetramerization process is initiated via interaction between the cytosolic domains and the stability of these tetramers is further enhanced via TM intra-domain interaction. The multimerization of tetramers, which is different than dynamic aggregation of GLUT1 reported previously (Pascual et al. 2008), occurs most likely via cooperativity among tetramers of modestly expressed GLUT1, as depicted in the model shown in Fig. 6. In this regard, genetic mutations that destabilize native conformation and re-establish non-native interactions, as detected by our modeling and aggregation prediction analyses, could drastically affect the folding of intracellular helices (ICH) that interact with the cytosolic parts of the TM domains via extensive polar interactions (Deng et al. 2014; Kapoor et al. 2016). Because many mutations derived from GLUT1-DS map to residues that mediate the inter-domain interactions on the cytosolic side, the misfolding in this region may result in aggregation of GLUT1 subunits or misfolded oligomeric complexes. It is also possible that TM9, which is included in predicted aggregation-prone regions, could determine an initial aggregation of GLUT1 tetramers. However, genetic mutations might increase the aggregation potential by triggering redundant interactions, further destabilizing GLUT1 conformation (Qin et al. 2013).

In addition to GLUT1 tendency to aggregate as a function of GLUT1-DS mutation, we would also like to propose how genetic mutations could affect transport mechanism of monomeric GLUT1 mutants, when expressed at the cell surface. In this regard, at least two kinetic models (the simple carrier and the fixed-site carrier) can be considered. The simple carrier, which was proposed to alternate between exofacial and endofacial orientations (Vollers and Carruthers 2012), may not be able to bind sugar and undergo a conformational change in GLUT1-DS. For instance, mutations at positions N34 or T310 that could disorganize the outer face of GLUT1 leading to improper binding of sugar to the exofacial face and compromising sugar uptake. Significantly reduced membrane association of S66F mutant also points to the same effect. G76D and G91D would be considered to lock the transporter in a rather rigid conformation that is not flexible to allow alternating orientations. R126 and E146 mutations, located quite close to the outer and inner face of the transporter, respectively, would destabilize the meshwork of interactions leading to improper binding of sugar to both exofacial and endofacial faces of the transporter. Furthermore, mutations of R218, K256, and R333 would restrict the movement of intracellular IC1 and 2, impairing endofacial orientations and sugar translocation.

Considering the fixed-site carrier model (Vollers and Carruthers 2012), where GLUT1 simultaneously exposes endofacial and exofacial sugar binding sites and a large central cavity to allow exchange of bound sugar with cavity sugar (known as geminate exchange), mutation of N34, T310, or S66 would lead to improper binding of sugar to the exofacial face, thereby inhibiting geminate exchange and sugar translocation. Mutations of G76, G91, E146, and R218 would impair the flexibility of the carrier at the endofacial face thereby restricting the exchange of bound sugar with cavity sugar. R126 mutation would potentially lead to improper binding of sugar to exofacial face. Mutations of R333 or K256 would also be expected to impair the geminate exchange and sugar translocation process.

This study investigates the structural consequences of GLUT1-DS mutations and their effects on function that may help us understand the structural basis of sugar transport. Lastly, GLUT1 mutants with a higher propensity to aggregate might exhibit stronger potential to partition into membranes and cause pathogenesis. The homeostasis of aggregated proteins may trigger sporadic and familial disease (Qin et al. 2013), similar to what has been described for many aggregated proteins in diseased states.

## Electronic supplementary material

Below is the link to the electronic supplementary material.

Supplementary material 1 (PPTX 1112 kb)

Supplementary material 2 (DOCX 46 kb)
