# Supplementary material for: Mechanistic Insights into Protein Stability and Self-aggregation in GLUT1 Genetic Variants Causing GLUT1-Deficiency Syndrome
Source: J Membr Biol. 2020 Feb 5;253(2):87–99. doi: 10.1007/s00232-020-00108-3 (PMC7150661; doi:10.1007/s00232-020-00108-3)
Supplement: Supplementary file 1 — Supplementary material 1 (PPTX 1112 kb) [file 232_2020_108_MOESM1_ESM.pptx]

## Slide 1
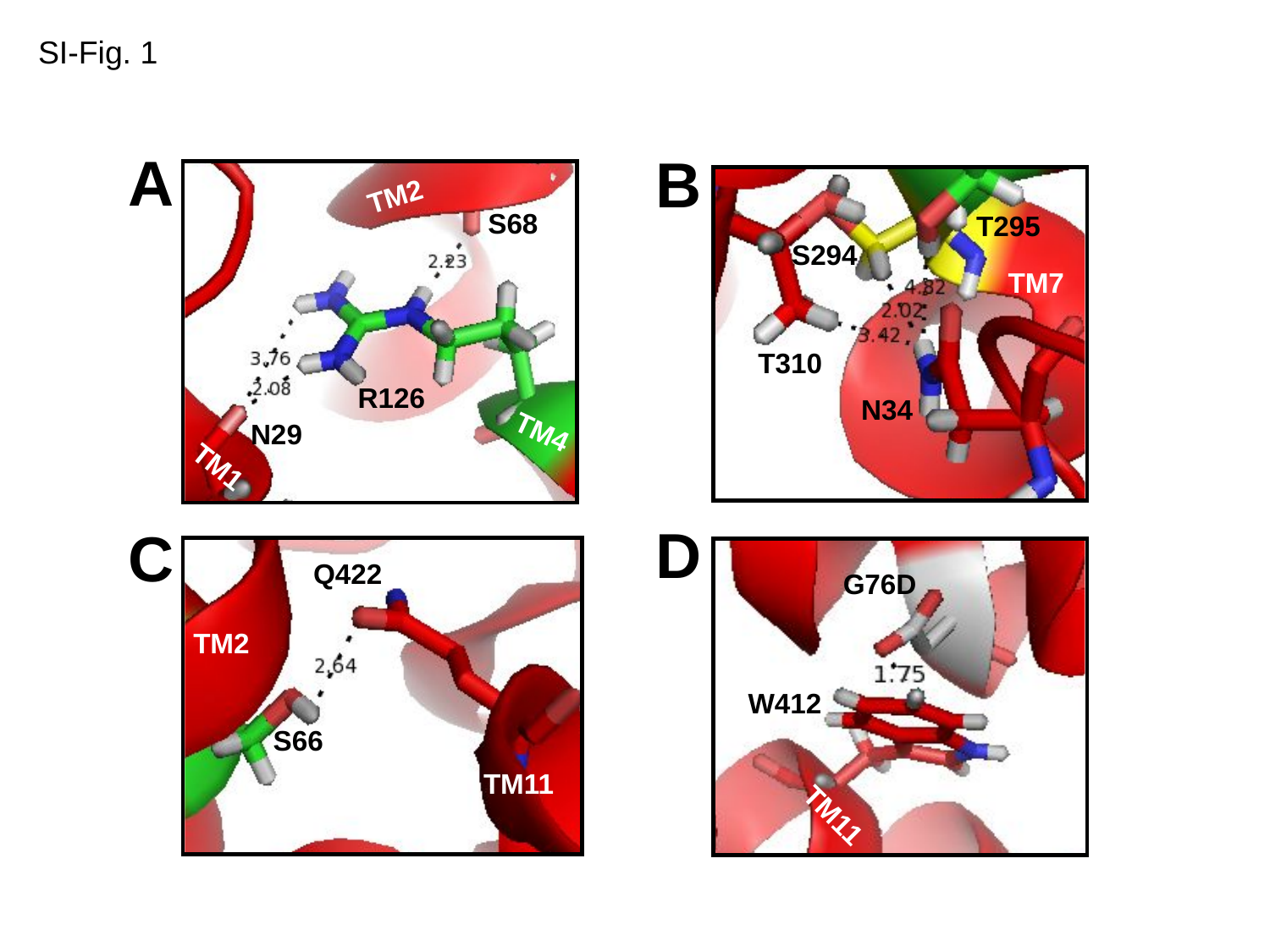

SI-Fig. 1
A
B
TM2
S68
T295
S294
TM7
T310
R126
N34
TM4
N29
TM1
D
C
Q422
G76D
TM2
W412
S66
TM11
TM11

## Slide 2
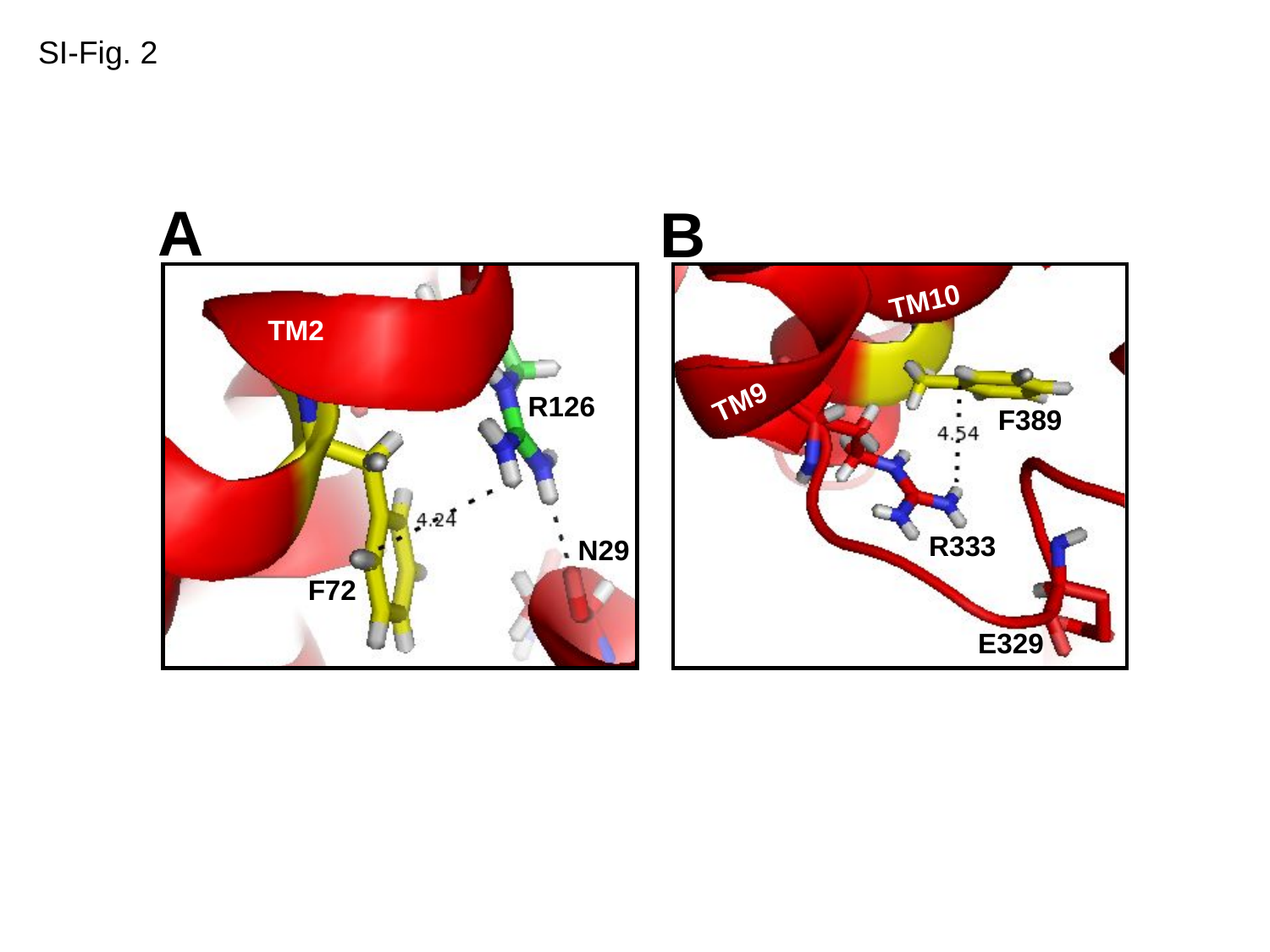

SI-Fig. 2
A
B
TM10
TM2
TM9
R126
F389
R333
N29
F72
E329

## Slide 3
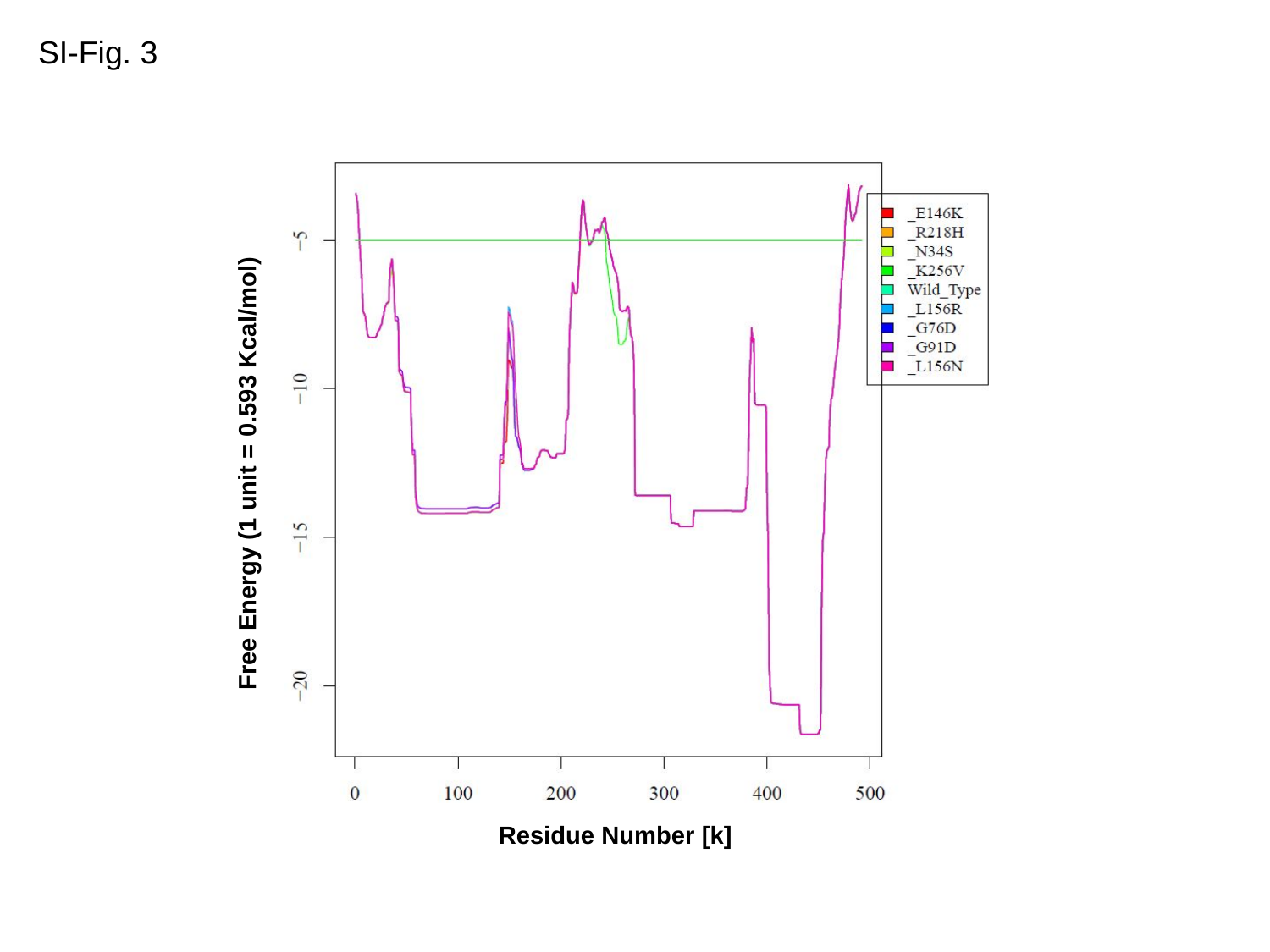

SI-Fig. 3
Free Energy (1 unit = 0.593 Kcal/mol)
Residue Number [k]

## Slide 4
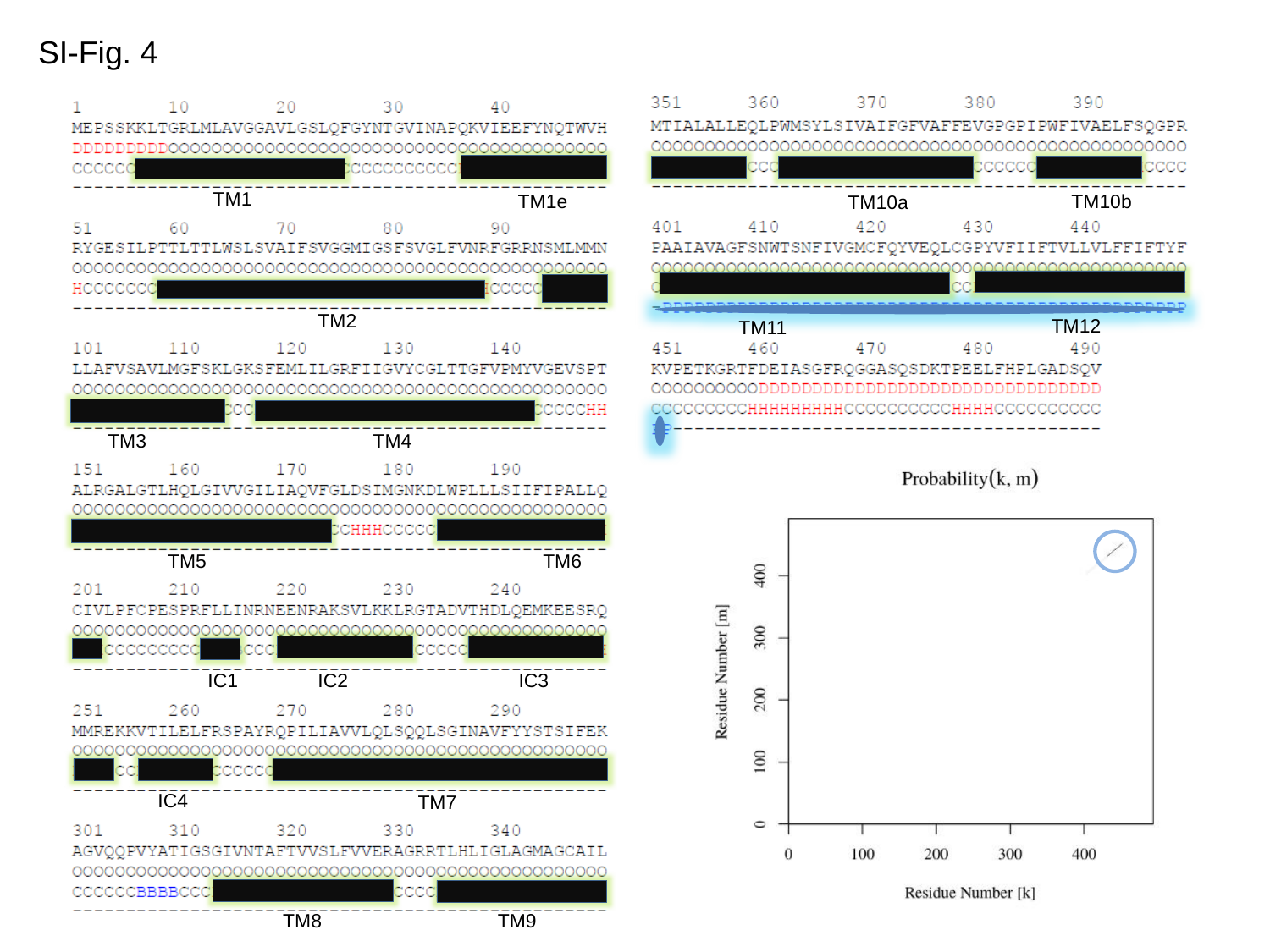

SI-Fig. 4
TM1
TM1e
TM10b
TM10a
TM2
TM12
TM11
TM3
TM4
TM5
TM6
IC1
IC2
IC3
IC4
TM7
TM9
TM8

## Slide 5
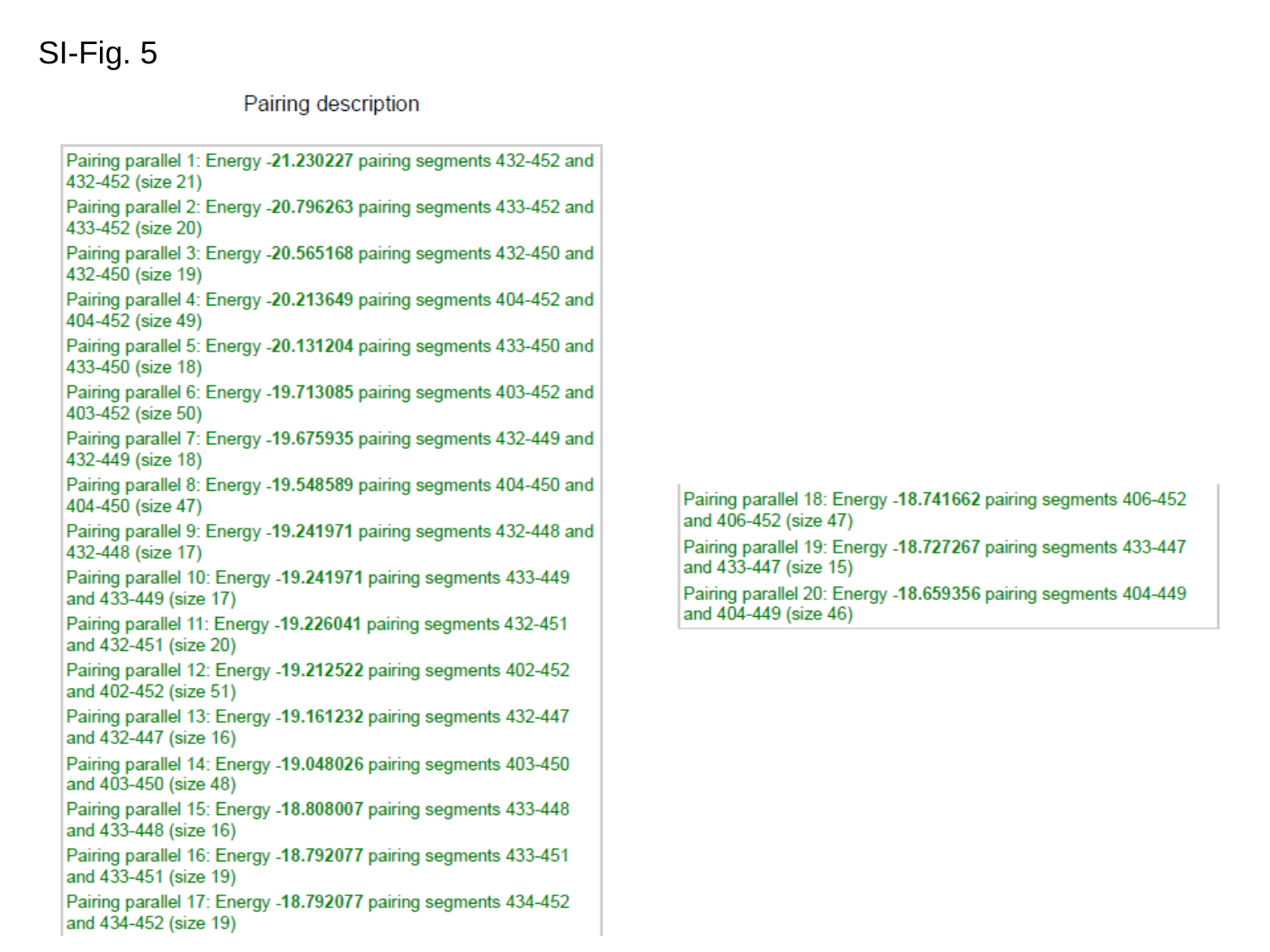

SI-Fig. 5

## Slide 6
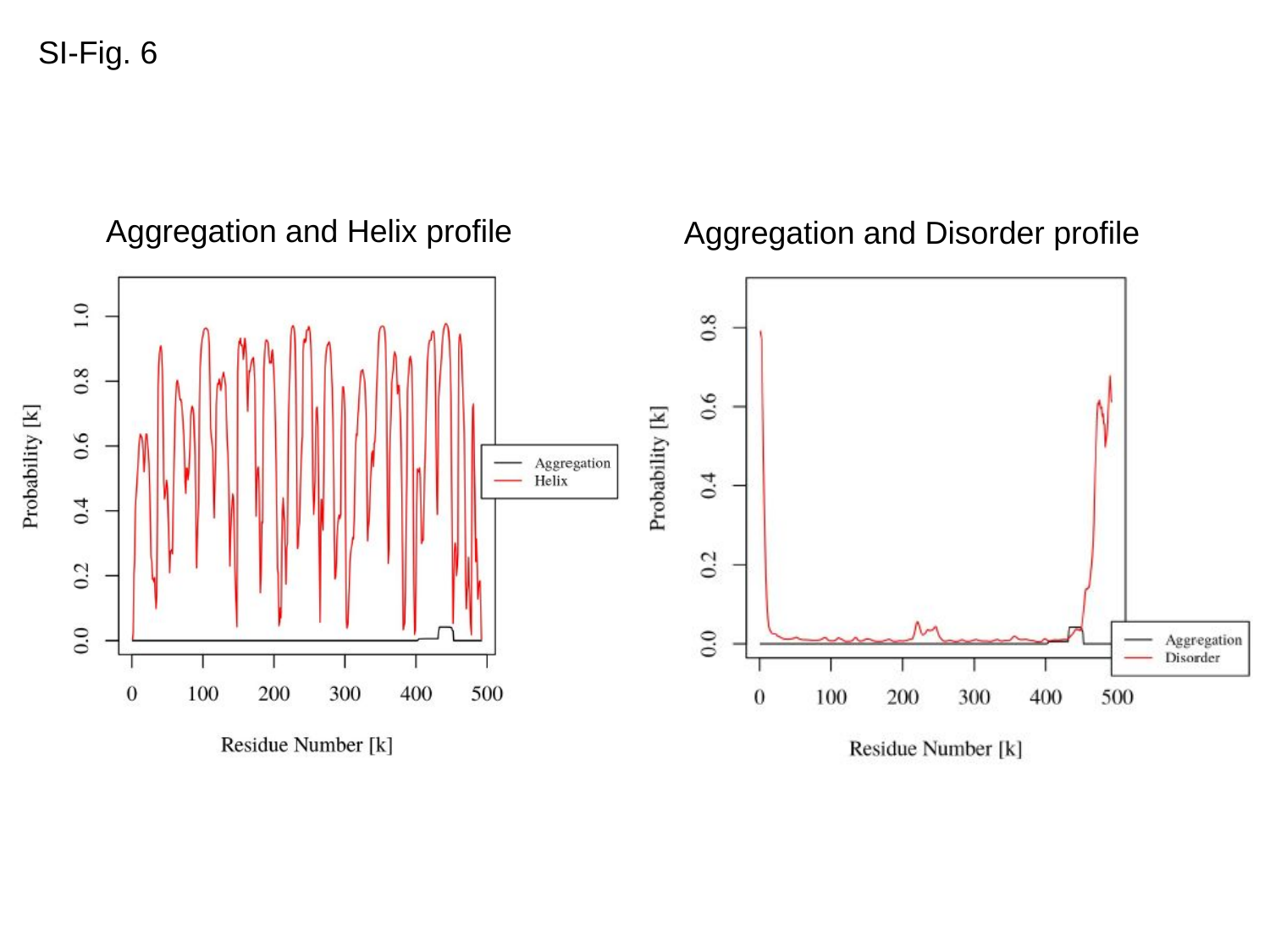

SI-Fig. 6
Aggregation and Helix profile
Aggregation and Disorder profile
