# Supplementary material for: Mechanistic Insights into Protein Stability and Self-aggregation in GLUT1 Genetic Variants Causing GLUT1-Deficiency Syndrome
Source: J Membr Biol. 2020 Feb 5;253(2):87–99. doi: 10.1007/s00232-020-00108-3 (PMC7150661; doi:10.1007/s00232-020-00108-3)
Supplement: Supplementary file 2 — Supplementary material 2 (DOCX 46 kb) [file 232_2020_108_MOESM2_ESM.doc]

**Supplementary Information - Figure legends**

**SI-Fig. 1.** Modeling of R126 (A), T310 (B), S66 (C), and G76D (D) to depict native and novel interactions. The bond lengths and distance between the two side-chains calculated in PyMol are shown.

**SI-Fig. 2.** Modeling of R126 (A), T310 (B), S66 (C), and G76D (D) to depict native and novel interactions. The bond lengths and distance between the two side-chains calculated in PyMol are shown.

**SI-Fig. 3.** Aggregation free energy profiles of WT vs. mutants N34S, G76D, G91D, E146K, L156R/N, R218H and K256V.

**SI-Fig. 4.** Amino acid sequence of WT-GLUT1. The prediction of parallel aggregates (P) formed among residues 402-452 are highlighted in blue. The probability of aggregation among pairs (denoted by *k* and *m*) are also represented. The helical prediction (highlighted in green) by PASTA 2.0 agrees well with GLUT1 structure and sequence.

**SI-Fig. 5.** The pairing description of residues 402-452 for WT-GLUT1. Parallel aggregation (pairing parallel) of each segment is predicted based on free energies. The total number of 20 aggregates or pairing segments of varying sizes that occurred in the region between residues 402 and 452 are shown.

**SI-Fig. 6.** The linear probabilities of aggregation *vs.* helix profile and aggregation *vs.* disorder profile of WT-GLUT1.
